# To change or not to change - translating and culturally adapting the paediatric version of the Moral Distress Scale-Revised (MDS-R)

**DOI:** 10.1186/s12910-017-0176-y

**Published:** 2017-02-20

**Authors:** Margareta af Sandeberg, Marika Wenemark, Cecilia Bartholdson, Kim Lützén, Pernilla Pergert

**Affiliations:** 1 Department of Women’s and Children’s Health, Karolinska Institutet, SE-171 76 Stockholm, Sweden; 2 Children’s and Women’s Health, Karolinska University Hospital, SE-171 76 Stockholm, Sweden; 3 Department of Medicine and Health Sciences, Linköping University, SE-581 83 Linköping, Sweden; 4 Centre for Healthcare Development, Region Östergötland, SE-581 85 Linköping, Sweden

**Keywords:** Cognitive interviews, Cultural adaptation, Difficult ethical situations, Healthcare professionals, Moral distress, Paediatric cancer care, Questionnaire, Respondent satisfaction, Translation procedure

## Abstract

**Background:**

Paediatric cancer care poses ethically difficult situations that can lead to value conflicts about what is best for the child, possibly resulting in moral distress. Research on moral distress is lacking in paediatric cancer care in Sweden and most questionnaires are developed in English. The Moral Distress Scale-Revised (MDS-R) is a questionnaire that measures moral distress in specific situations; respondents are asked to indicate both the frequency and the level of disturbance when the situation arises. The aims of this study were to translate and culturally adapt the questionnaire to the context of Swedish paediatric cancer care. In doing so we endeavoured to keep the content in the Swedish version as equivalent to the original as possible but to introduce modifications that improve the functional level and increase respondent satisfaction.

**Methods:**

The procedure included linguistic translation and cultural adaptation of MDS-R’s paediatric versions for Physicians, Nurses and Other Healthcare Providers to the context of Swedish paediatric cancer care. The process of adjustment included: preparation, translation procedure and respondent validation. The latter included focus group and cognitive interviews with healthcare professionals in paediatric cancer care.

**Results:**

To achieve a Swedish version with a good functional level and high trustworthiness, some adjustments were made concerning design, language, cultural matters and content. Cognitive interviews revealed problems with stating the level of disturbance hypothetically and items with negations caused even more problems, after having stated that the situation never happens.

**Conclusions:**

Translation and cultural adaptation require the involvement of various types of specialist. It is difficult to combine the intention to keep the content as equivalent to the original as possible with the need for modifications that improve the functional level and increase respondent satisfaction. The translated and culturally adapted Swedish MDS-R seems to have equivalent content as well as improved functional level and respondent satisfaction. The adjustments were made to fit paediatric cancer care but it could be argued that the changes are relevant for most areas of paediatric care of seriously ill patients.

## Background

Working with paediatric cancer care involves continuously facing ethically difficult and complex situations that often lead to value conflicts in the team, and sometimes with the families, about what is best for the child [1], resulting in possible experience of moral distress [2, 3].

### Moral distress

Moral distress was originally described by Jameton [4] as arising when one knows the right thing to do, but perceived or prevailing institutional constraints prevent the “right” action. In a later publication, Jameton [5] pointed out that moral distress could be distinguished from moral dilemma; i.e., not knowing which action is right, since each alternative action is perceived to involve a value conflict. However, O’Donnell includes value conflicts as a cause of ethical stress [6]. Lutzen and Kvist [7] proposed a conceptual distinction between moral distress and moral stress. Moral distress encompasses negative psychological components, while moral stress widens the perspective to include physiological consequences of a moral demand, which also include positive consequences such as moral reflection [7]. In this study we will use the term moral distress in the following sense: Moral distress occurs in situations when someone has a perception of what is ethically right, but cannot act accordingly [8–10] and also when someone does not know what is ethically right, but has to make a decision [6]. Thus, moral distress also includes situations of moral dilemma, as a consequence of a moral demand.

### Previous research

Situations that have been described to generate moral distress among nurses are related to: providing unnecessary/futile treatment [2, 3, 11]; prolonging the dying process through aggressive treatment [2, 3]; treating symptoms and fearing that this may hasten death [11]; honest/dishonest communication and different views about truth telling [2, 3, 11]; and having to carry out painful procedures against children’s will [2]. Many nurses and physicians have acted against their conscience in the context of end-of-life care of children [12]. Physicians have in some studies been reported to experience lower levels of moral distress than nurses [13], though a recent paediatric study found that levels of moral distress were highest among physicians [14].

An individual’s experience of moral distress is dependent on several factors: internal constraints such as perceived powerlessness and increased moral sensitivity [8, 10] and external constraints such as inadequate communication among team members, differing inter-professional (e.g. Registered Nurses (RN) to Physicians) or intra-professional (e.g. RN to RN) perspectives as well as inadequate staffing and increased turnover [8, 10]. Levels of moral distress are also influenced by the ethical climate [15]. A Swedish study concluded that nurses’ perceptions of a more positive ethical climate were related to fewer reports of morally distressing situations [16]. Research among healthcare professionals revealed that those with longer experience reported higher levels of moral distress due to the so-called “crescendo effect”, that is, the negative build-up over time after repeated experiences of moral distress [17].

Moral distress has been shown to be related to burnout, low job-satisfaction and intention to leave a clinical position [18, 19], and thus to be a contributory factor to high turnover of healthcare professionals. Preventing moral distress and consequently decreasing exhaustion and burnout is extremely important for promoting job satisfaction [20, 21]. It is therefore important to explore and understand perceptions of moral distress and experiences of specific situations that augment it.

### Measuring moral distress

There is an ongoing discussion about the possibility of measuring moral distress. An example of an initiative in this field is the Work Related Moral Stress Scale [15], available in Swedish, which measures symptoms of stress. One problem with this scale could be for healthcare professionals to distinguish between moral, emotional, private and work-related strains as the cause of stress. It might be preferable to use more specific measures, rating levels of disturbance in situations that have been found to generate moral distress. One of the most commonly used questionnaires for measuring moral distress in specific situations is the Moral Distress Scale (MDS) [8]. For example, the paediatric version of the MDS has been used in paediatric oncology in Italy [2]. The MDS has been revised (MDS-R) in order to include more possible root causes of moral distress, including: clinical situations, internal constraints and external constraints, and different versions have been created for physicians, nurses and other care providers in both paediatric and adult settings [10]. The MDS-R was recently used in a sizeable American study of three hospitals with sixty different paediatric specialities [14]. Others have either adapted MDS or developed other scales or questionnaires to measure concepts similar to moral distress in other cultures and populations [22–28]. An unfortunate result of this productive and worthwhile work is the lack of uniformity or agreement on a reliable and valid measure of moral distress for use across disciplines and settings, including paediatric settings.

Research on moral distress is lacking in paediatric cancer care in Sweden and most questionnaires, including the MDS-R, have been developed in English. In order to make the questionnaires suitable in the Swedish context they have to be translated and culturally adapted by a rigorous method in order to produce equivalency between the original and the new versions [29]. Not only do the questionnaires have to be translated well linguistically, but their cultural adaptation is also highly important to maintain the validity of their content at a conceptual level between cultures [29]. Another important aspect is the need for the questionnaires to have a good functional level in terms of the respondent’s cognitive process of understanding and answering the questions [30]. A good functional level is important to reduce response errors and make respondents feel competent enough to respond to the questions. All questionnaires and surveys rely on respondents’ willingness to answer the questions. Trust, competence and autonomy have been found to be three necessary conditions for achieving intrinsic motivation in general [31]. Questionnaires with a good functional level and trustworthiness are therefore important for enhancing respondents’ motivation, which may improve response quality as well as respondent satisfaction [32].

The aims of this study were to translate and culturally adapt the paediatric MDS-R to the context of Swedish paediatric cancer care. These aims also included keeping the content in the Swedish version as equivalent to the original as possible while also introducing modifications that improve the functional level and increase respondent satisfaction.

## Methods

In this study, MDS-R was translated and culturally adapted to Swedish paediatric cancer care.

### The moral distress scale

The MDS was originally designed to assess moral distress among nurses working in intensive care units [8]. MDS-R consists of 21 items [10, 13] and has demonstrated adequate reliability and construct validity [10]. The items are phrased as statements and for each statement, the respondents are asked to indicate, on a 0–4 Likert scale, both the frequency (how often the situation arises) and the level of disturbance (intensity) when the situation arises. The respondents are also asked to indicate intensity, even if they have not experienced a situation. In this study, to facilitate data collection, the three paediatric versions, for Physicians, Nurses and Other Healthcare Providers, of the MDS-R [10] were combined into a single questionnaire. Some items are formulated slightly differently in the three versions to reflect the respective groups’ specific responsibilities and duties.

### Procedures

The procedure included linguistic translation and cultural adaptation of MDS-R’s three paediatric versions to the context of Swedish paediatric cancer care. During preparations for the process and reading the literature, two rigorous guidelines turned out to be relevant and suitable [29, 33] and various steps from both of them were therefore used. Thus, the overall procedure follows the scientific structure that permeates both guidelines.

#### Preparation [33]

First, the original designer of the MDS, Dr Corley, was contacted and she recommended us to use the revised version, referring to Dr Hamric. Permission was obtained from Dr Hamric to translate and culturally adapt the MDS-R to Swedish paediatric cancer care and it was agreed that data should be shared with Dr Hamric and Dr Corley in order to further psychometric testing of the instrument.

#### Initial translation [29]

The translation of MDS-R from English into Swedish was done in two versions by translators with different profiles, independently of each other. Translation 1 was done by the first (MafS) and last (PP) authors, who are specialists in paediatric cancer care and ethics, i.e., with good knowledge of the context being examined. Translation 2 was done by a certified translator with no previous knowledge of the context, focusing to a greater extent on different meanings in the two languages. Furthermore, the translators kept notes with comments about uncertainties and questionable phrases as well as arguments for their choices.

#### Synthesis of the translations [29]

The synthesis of the two translated versions was performed by a review group (*n* = 6) that, besides the translators of versions 1 and 2, consisted of additional expertise in ethics and paediatrics, including the 3^rd^ and 4^th^ authors. The two translated versions were compared with the original questionnaire in order to achieve consensus and to synthesize the two versions into one. For example the translation of the word “distress” was explored. Each discussed issue and the final solution were documented in written notes.

#### Cognitive debriefing [33]

The next step was to test the synthesized version’s face validity, item relevance and respondent satisfaction. This was done in two rounds, consisting of focus groups and cognitive interviews. The focus group (*n* = 14) in the first round included consultant nurses in paediatric oncology and consultant nurses for children with brain tumours. Thereafter cognitive interviews were performed, as described by Collins [34], with healthcare professionals in paediatric oncology, including: RNs (*n* = 6) and physicians (*n* = 3) with ethical expertise. The focus group (*n* = 8) in the second round included experienced paediatric oncology nurses. The cognitive interviews were performed with RNs (*n* = 3) and nurse assistants (*n*= 4) in paediatric cancer care but without ethical expertise. The cognitive interviews were performed by the first and second authors (MafS and MW), of whom the latter has extensive experience of cognitive interviews. The participants were informed that the researchers were primarily interested, not in the actual answers but in the participants’ experiences of understanding and answering the questions. The first part of the interview was performed as a think-aloud where the participants were encouraged to verbalize their thoughts during the process of responding to the questionnaire. During the think-aloud the interviewer took notes discretely and intervened as little as possible. In the next step the interviewer asked a few planned probes about the items and followed this up with spontaneous probes, e.g., issues that had caused comments and concerns during the think-aloud [34].

#### Review of cognitive debriefing [33]

All modifications throughout the process were discussed with the review group, enhanced with the second author (MW), who is a researcher specialized in questionnaire design. Based on the results from the focus group and the cognitive interviews in the first round, modifications were made. For example, some items required reformulation to suit the legal and healthcare systems. Furthermore, participants in the focus group suggested new relevant items, which led to continuous in-depth discussions in the review group (*n* = 5) concerning the balance between changing and not changing. Already in the first round a problem with hypothetical thinking emerged in some situations and this was dealt with in the second round. Members of the review group (*n* = 3) had several meetings during and after the second round, discussing and making modifications. For example, discussions concerned how to handle the added questions, with the intention of minimizing changes to the original MDS-R.

#### Back translation [29]

To ensure content equivalence and perform a validity check between the original and the Swedish version, the latter was translated back into English by a native English-speaking certified translator. As this translator had not seen the original English version, the back translation could be used to ensure that the item content of the Swedish MDS-R was the same as in the original English version.

#### Review of the back translation [33]

The back-translated version and the original questionnaire were compared by the review group (*n* = 5) in order to discuss identified differences in wording, concepts and possible alterations, with the objective of achieving a mutual decision and agreement on necessary revisions. The review group discussed, for example, that the original MDS-R uses four different words with similar conceptual meanings and decided that those could all be translated into a single Swedish word.

## Results

The main goals of the translation and cultural adaptation of the MDS-R were to retain the conceptual equivalence of the original questionnaire but at the same time achieve a Swedish version with a good functional level and high trustworthiness. The adjustments made concerned design, language, cultural aspects and content.

### Adjustments to questionnaire design

Adjustments to the design of the questionnaire were made to facilitate data collection but primarily to improve respondent satisfaction. The original questionnaire starts with instructions and a definition of moral distress. The definition was excluded because, before answering, the participants in the cognitive interviews tended to try to figure out whether the nature of the disturbance in the described situations was moral distress according to the definition. Furthermore, in the original version the introduction informed participants to “indicate how frequently you experience each item described and how disturbing the experience is for you. If you have never experienced a particular situation, select “0” (never) for frequency.” This led to participants going up and down between the items and the instruction and therefore, in the Swedish version this text was moved from the introduction to the headings of the two columns. Thus the instruction for each column was changed from “Level of disturbance” to “How would this situation affect you?” and from “Frequency” to “How often have you experienced this situation?” Consequently, the verb in the beginning of statements, for example “provide”, was changed to show activity and since the Swedish language lacks gerunds the word “to” was added, for example “to provide”, synonymous with “providing” in English. Changing the wording from “how disturbing the situation is” to “how disturbing the situation would be” also made it easier for participants who had never experienced the situation to answer.

Moreover, the cognitive interviews revealed problems with stating the level of disturbance hypothetically. Some of the participants who said they had never experienced a situation responded hypothetical disturbance as intended (“I haven’t experienced it, but it would be terrible if it happened…”), but others reported no disturbance (“I haven’t experienced it so it hasn’t disturbed me…”). These two ways of reasoning varied between participants and some participants switched between the right and wrong reasoning on different items (sometimes even several times). Thus, the cognitive interviews clearly showed that participants sometimes made mistakes (reporting 0 disturbance since it did not happen) and others alternated between right and wrong reasoning. Situations they had not experienced, but could imagine as possible, would more often lead to correct reasoning and a hypothetical answer about the level of disturbance. However, situations that participants had difficulty in even imagining as possible would more often lead to incorrect reasoning and an answer of no disturbance. The respondents also expressed uncertainty about how to answer correctly, leading to frustration and fear of making mistakes.

The two items with negations caused even more problems for respondents who stated that the situation never happens. For example, in one item the statement is “To avoid reporting to your superior …. medical error”. The negation in the formulation of the item caused hesitation, especially when using the response option “never”, which meant that they answered “I have never avoided reporting”. The cognitive interviews also revealed that the participants had often experienced the opposite of what was described, that is, they had reported a co-worker and it affected them. Thus, when using the response option “never” avoided reporting, instead of considering the level of disturbance from avoiding to report if it occurred, some asserted they had written deviation reports and how much that disturbed them. For this reason, in the Swedish MDS-R the respondents are asked to indicate the level of disturbance (intensity) when the situation arises *before* stating the frequency (how often the situation arises). Participants with experience of the situation were unaffected by this change and still based their answers on their experiences. For participants without experience of the situation, the change helped them to think hypothetically, as was intended. It also helped to avoid the problem with answering about situations that included negations.

To obtain a joint questionnaire that would be relevant for all the healthcare professionals it was necessary to keep or formulate four items differently in the merged version to reflect differences in responsibility and possible experience between the three professions. Two of these items were formulated in one way for RN and nurse assistants and in another way for physicians, while the other two were formulated in one way for RN and physicians and another way for nurse assistants. To make it easier for the respondent to identify the profession-specific part of the items, these were highlighted in bold type. The cognitive interviews indicated that the profession-specific-items in the merged version did not cause any problems for the participants.

### Linguistic adjustments

Linguistic adjustments were made to keep close to the meaning of the original items; four examples are given below. In the present context the translation of the word “distress” is the Swedish word for pain or anguish (Swedish: “kval”) but this is an old-fashioned word that is no longer commonly used, so “distress” was translated into the Swedish word for stress.

Instead of using the exact translation of a concept, a word that would be used in the Swedish context was chosen. For example, “assist a physician” is used in one original item; in cognitive interviews, both nurses and physicians stressed that they work in teams and therefore the expression “work with” was considered more appropriate (“assist” is used predominantly when assisting a surgeon). Thus, “assist” was changed to “work with”.

Another example is the concept “ignore”. In cognitive interviews, participants perceived that the word ignore alluded to a bad attitude. Therefore, the Swedish translation of the word “ignore” (Swedish: “ignorera”) was changed to “shut eyes to” (Swedish: “blunda för”), equivalent to the meaning of the original item.

Another linguistic change was that the word “despite” (Swedish “trots”) was used in four items instead of “even though”, “when” or “that”. This was done to make the value conflict in the situation more obvious.

### Cultural adjustments to a Swedish context

Cultural adjustments to a Swedish context were made to ensure that items were relevant to the structure and organisation of Swedish society and healthcare. For example, the original wording of one item was “Provide less than optimal care due to pressures from administrators or insurers to reduce costs”. Swedish healthcare is publicly funded and normally insurers do not have an impact on the financial aspects of healthcare. Thus, “administrators or insurers” was changed to “management”.

The original three versions of the questionnaire target three different professional groups: physicians, nurses and other healthcare providers. To match the Swedish context, in the Swedish version the targeted professional group was altered to include physicians, RNs and nurse assistants. Because nurse assistants are a large and important group it was not considered appropriate to label them as “other”, particularly as the aim was to target all healthcare professionals who have experienced the described situations, in close patient care.

### Cultural adjustments to paediatric cancer care

Cultural adjustments to paediatric cancer care were made to ensure that items were relevant to this context. For example, the original wording of one item was: “Increase the dose of sedatives/opiates for an unconscious child that I believe could hasten the child’s death”. The concept “unconscious” was deleted because normally patients in paediatric cancer care units are not unconscious. In addition, in this item “Increase the dose … that I believe could hasten the child’s death” was perceived by some participants as an issue concerning euthanasia; to prevent this, the words “despite that” were added, which also presents the situation’s moral dilemma more clearly.

### Adjustments in terms of content

Cognitive interviews revealed that some specific situations generating moral distress were not captured in the original questionnaire. Therefore, five items were added at the end of the questionnaire in order to achieve relevance with respect to the participants’ experiences while retaining the content equivalence of the original questionnaire.

A frequent comment, while answering an item about providing less than optimal care, was that it was often related to an inability to communicate satisfactorily with the child and the family, due to a lack of time. Thus, an item was added to cover this possible situation.

The item about the family’s wishes concerning treatment led to discussions about difficult parents and a general tendency to have unrealistic expectations as a common cause of moral distress. This aspect was covered by an added item.

While answering an item concerning the family’s request not to talk about death with a dying child, participants’ comments showed that not talking about death with dying children was an important issue and a source of distress regardless of the reason. Therefore, the item “To not talk about death with a dying child, despite that you think it is necessary” was added.

Another added item concerned performing painful/unpleasant procedures on school-age children who resist such treatment. This was identified in cognitive interviews as situations that could generate moral distress, when participants were answering an item on performing tests that were perceived as unnecessary and another item about students performing painful procedures.

During the assessment of the final questionnaire it became obvious that one of the two main definitions of moral distress, as a consequence of not knowing what is ethically correct but having to make a decision, was not captured in the original MDS-R and was consequently formulated as the last added item.

## Discussion

This paper describes linguistic translation and cultural adaptation of the MDS-R to fit Swedish paediatric cancer care and to improve the functional level and achieve respondent satisfaction, using well-known guidelines and methods for the scientific process, described in the literature [29, 33–35]. Several linguistic and culture-specific concepts were changed in order to achieve equivalence between the original source and the Swedish version of the questionnaire. Beaton et al. argue, using a scenario scale adapted from Guillemin and colleagues in 1993, that when a questionnaire is to be used in another country and another language, as is the case in this study, a translation needs to be accompanied by cross-cultural adaptations [29, 36].

The initial aim was to keep the content in the Swedish version as equivalent to the original as possible but during the respondent validation it became obvious that modifications were needed to improve the functional level and increase respondent satisfaction. Not just to make it easier for the respondents but also to improve the cognitive answering process, promote correct understanding of questions and further high-quality answers. There is an excessive reliance on validated questionnaires, although many established scales were developed without respondent focus. Usually a lot of effort has been devoted to the content (what to ask) but less to the design of questions (how to ask) and we therefore performed the cognitive debriefing according to Wild [33] before the back translation of the questionnaire. Today there is a stronger emphasis on taking respondents’ perspective into account when developing questions and scales. Methods, such as cognitive interviews, have the potential to reveal problems with old questionnaires that cannot be neglected if one is to get valid answers. It is therefore extremely important to examine a questionnaire thoroughly before using it and to continue evaluating its performance.

In the original MDS-R, the respondents are asked to indicate the frequency of a situation (how often it arises) and then its intensity (level of disturbance) when it arises, even if they had indicated that the situation never occurs. Thus, participants who answered that the situation never occurs were expected to answer hypothetically how much it would affect them if it did happen. This hypothetical thinking resulted in participants making mistakes and being uncertain about how to answer. Hypothetical thinking has previously been described as being difficult because it requires thinking through adversative situations [37]. One could discuss whether answering a questionnaire about experiences should require hypothetical thinking. However, in choosing between asking those without experience to answer “level of disturbance” hypothetically or not at all, the first option was chosen in order to give each respondent opportunities to reflect on and answer all items, especially since the altered order made the response process less problematic for respondents without experience of a given situation.

Even established questionnaires may need to be revised as populations and language change over time. In order to yield motivation among respondents, which may improve response quality as well as respondent satisfaction, it is important that questionnaires have a good functional level and are trustworthy. Previous research has found that the relevance of a questionnaire’s content can have an impact on respondents’ motivation to respond in a thoughtful and trustworthy way [38]. In times of declining response rates, we argue that it is important for every researcher to use relevant and cognitively well-functioning questions to preserve public confidence in measurements and surveys. The contents and items of the MDS have been thoroughly discussed but we have not found any feasibility or cognitive evaluations. Some authors have commented on problems with respondent confusion about how to answer the intensity question in the event of zero frequency [2]. Even if they conclude that the confusion does not affect the distress score, it may be frustrating for respondents, as shown by the cognitive interviews in the present study.

The response process for the two items that included negations was even more complicated. All the participants in cognitive interviews reasoned inversely when deciding how to answer how much it affected them. Thus when they answered that they never “Avoid taking action …” or “Take no action …” they reasoned that they did take action and answered how much that affected them. To deal with this problem with the design, we decided to change the arrangement of the columns so that participants began by answering how a situation would affect them. It could be argued that this made all the questions hypothetical and one could say the modification was a major one. We consider, however, that it improved respondent satisfaction. Also Järemo and Arman [35] described difficulties with items with negations (“…gives me unhelpful advice”) when choosing the response option “never” ([35] p.6).

It has been suggested that mixing negative and positive wording in questionnaires is associated with disadvantages such as that respondents misinterpret the negative items [39]. The disadvantages described by Sauro and Lewis [39] could partly explain the difficulties with dual negations. Thus one could argue that the negative wordings should have been removed; on the other hand, others would argue that alternation has its advantages [40].

Another change to design was that the three versions of the questionnaire were merged into a single version while still having some items formulated differently for different target professions. The main reason for this decision was logistical – to print and distribute one and the same questionnaire for the whole team.

Pauly and colleagues criticized the longer, 38-item version of MDS on the grounds that the items’ formulations could trigger feelings of discomfort or anxiety among those who already recognize their moral distress [22]. Furthermore, they considered Jameton’s definition of moral distress to be limited because it emphasizes the inability of nurses to act due to institutional constraints and disregards other circumstances that are truly beyond a person’s control, such as individual and contextual constraints. Already Webster and Baylis suggested that the definition of moral distress should be extended to include distress that arises when personal judgements or mistakes prevent one from acting in accordance with one’s beliefs or satisfaction [41]. Moreover, the questionnaires used in research on moral distress are often designed for nurses and based on the original definition of moral distress [4]. MDS-R was modified to include more root causes of moral distress; however, we discovered that it did not cover moral distress as a consequence of uncertainty and decision-making in value conflicts, so it could be argued that it did not fully match physicians’ experiences [42]. The limitations of the longer version of the MDS cannot be remedied just by revising it into a shorter version. Focusing exclusively on the content (by shortening) does not solve the problem; a revision also has to deal with the functional level.

The Swedish word for stress was chosen in an attempt to capture the conceptual meaning of distress as well as possible. This could be viewed as a limitation because of the distinctions made in previous research and because the MDS-R does not specifically measure physiological or positive consequences of moral stress [7]. However, the respondents do answer how much the situations would affect them negatively, and this could include psychological and emotional as well as physiological consequences.

Five items have been added because they emerged as important and relevant for respondents and could not be ignored solely because of the need to adhere strictly to the definition of the concept of moral distress. To gain trustworthiness towards healthcare professionals it is important that the questionnaire is perceived as capturing the most important aspects from the respondent’s perspective. The Swedish MDS-R includes consequences of value conflicts according to one definition of moral stress [6]. However, one could argue that this is already included in the MDS-R because of the three root causes described by Hamric et al. [10]. The root cause ‘internal constraints’ includes self-doubt and lack of assertiveness [10], which could be applied to the added item “To decide on care/treatment when you are uncertain about what is right”. Furthermore, most of the added items could fit the root cause ‘clinical situations’. For example the statement about having to carry out painful procedures against the children’s will, previously reported from paediatric cancer care [1, 2], could fit with the clinical situation “disregard for patient wishes” [10]. Furthermore, to not talk about death with a dying child, previously described as a concern in transcultural paediatric cancer care [43], fits with the clinical situation “lack of truth-telling” [10]. To keep the MDS-R intact, the added items were placed at the end.

Changes were made to improve the questionnaire’s functional level, e.g., the rearrangement of the columns and the changes to items with negations. Adding profession-specific questions and items on relevance in the paediatric setting are examples of changes that were made to improve relevance and trustworthiness. A limitation of the present study was that only nurses were included in the focus groups. However, the primary aim of the focus groups was to test item relevance and this did not emerge as an issue in the cognitive interviews with physicians and nurse assistants. As a result of all the changes, we would argue that the questionnaire has an improved functional level in the new language and setting. The adjustments were made to fit paediatric cancer care, however, it could be argued that the changes are relevant for most areas of paediatric care of seriously ill patients. Further research is needed to establish the Swedish MDS-R’s psychometric qualities, such as reliability.

## Conclusions

Translation and cultural adaptation is a process that requires a considerable amount of time and the involvement of various specialists. Even established questionnaires may need to be revised. However, it is difficult to pursue the intention to keep the content as equivalent to the original as possible while also making modifications that improve the functional level and increase respondent satisfaction. The translated and culturally adapted Swedish MDS-R consists of 26 items in total, and includes the original 21 items with equivalent content, plus 5 added items. The translated and culturally adapted Swedish MDS-R seems to have equivalent content as well as improved functional level and respondent satisfaction.

## Abbreviations

MDS: Moral distress scale
MDS-R: Moral distress scale-revised
RN: Registered nurse
